# Role of Electronic Data Exchange in an International Outbreak Caused by *Salmonella enterica* Serotype Typhimurium DT204b

**DOI:** 10.3201/eid0807.010414

**Published:** 2002-07

**Authors:** Elizabeth A. Lindsay, Andrew J. Lawson, Rachel A. Walker, Linda R. Ward, Henry R. Smith, Fiona W. Scott, Sarah J. O'Brien, Ian S.T. Fisher, Paul D. Crook, Deborah Wilson, Derek J Brown, Hjordis Hardardottir, Wim J.B. Wannet, Helmut Tschäpe, E. John Threlfall

**Affiliations:** * Public Health Laboratory Service Laboratory of Enteric Pathogens, London, United Kingdom; † Public Health Laboratory Service Communicable Diseases Surveillance Centre, London, United Kingdom; ‡ County Durham and Darlington Health Authority, Durham, United Kingdom; § North Glasgow University Hospitals National Health Service Trust, Glasgow, Scotland; ¶ Landspitali University Hospital, Reykjavik, Iceland; ** National Institute of Public Health and the Environment, Bilthoven, the Netherlands; †† Robert-Koch Institut, Harz, Germany

**Keywords:** Salmonella, outbreak, elecronic data exchange

## Abstract

From July through September 2000, patients in five European countries were infected with a multidrug-resistant strain of *Salmonella* Typhimurium DT204b. Epidemiologic investigations were facilitated by the transmission of electronic images (Tagged Image Files) of pulsed-field gel electrophoresis profiles. This investigation highlights the importance of standardized protocols for molecular typing in international outbreaks of foodborne disease.

## The Study

From July through September 2000, patients in five European countries (England, Scotland, Germany, the Netherlands, and Iceland) were infected with a strain of *Salmonella enterica* serotype Typhimurium definitive phage type (DT) 204b; the strain was resistant to ampicillin (A), chloramphenicol (C), gentamicin (G), kanamycin (K), streptomycin (S), sulphonamides (Su), tetracyclines (T), trimethoprim (Tm), and nalidixic acid (Nx). The strain also had decreased susceptibility to ciprofloxacin (Cp_L_), with an MIC by E-test of 0.38 mg/L (1,2). Over 350 laboratory-confirmed cases were recognized. Epidemiologic investigations implicated shredded lettuce as the vehicle of infection (1).

Isolates from patients in Iceland, the Netherlands, and Scotland were referred to the England and Wales Public Health Laboratory Service (PHLS) Laboratory of Enteric Pathogens for phage typing and antibiogram analysis. These isolates were compared with those from a concurrent outbreak of multiresistant *S.* Typhimurium DT204b in northeastern England and from patients returning to England and Wales after visiting other European countries. A panel of seven isolates from outbreaks in England, Scotland, Iceland, and the Netherlands and from patients returning to the United Kingdom after visiting Greece, Germany, and the Netherlands were further characterized by a variety of molecular techniques, including plasmid profile typing, pulsed-field gel electrophoresis (PFGE), fluorescent amplified fragment-length polymorphism fingerprinting (FAFLP), and integron typing; specific resistance genes were characterized by polymerase chain reaction (PCR) and the mutation conferring decreased susceptibility to ciprofloxacin was identified by a LightCycler (Roche Diagnostics Ltd., Lewes, U.K.) *gyrA* mutation assay (GAMA) (3). Isolates from Germany and Scotland were typed independently with the same phenotypic methods as those used in the Laboratory of Enteric Pathogens and also by plasmid profile typing and PFGE. To facilitate epidemiologic investigations, PFGE Tagged Image Files (TIFs) of banding patterns of isolates from England, Scotland, and Germany were exchanged electronically.

Conjugation experiments were performed at both 28°C and 37°C by using a rifampicin-resistant strain of *Escherichia coli* (strain 20R764) as the recipient. Resultant plasmids were classified by incompatibility. DNA was extracted from transconjugants by using a DNeasy tissue kit (Qiagen Ltd., Crawley, U.K.). For plasmid profile analysis, plasmids were resolved by electrophoresis at 110 V for 3 hours in 0.8% wt/vol agarose. Oligonucleotide primers synthesized by MWG-Biotech UK Ltd. (Milton Keynes, U.K.) were used to detect the antibiotic resistance genes *aad*A2, *bla*_CARB-2_, *bla*_TEM_, *sul1*, *tet*A (class A), *tet*A (class G), *tet*A (class B), and integrons in both wild-type strains and *E. coli* K12 transconjugants. The nucleotide sequence of these primers and the corresponding temperature profiles for amplification have been described (4). GAMA, designed to detect three different *gyrA* mutations, was performed in a LightCycler instrument under previously described reaction components and conditions (3). For PFGE, agarose plugs were prepared by the method of Powell et al. (5) with 2% chromosomal grade agarose (Bio-Rad Laboratories, Hemel Hempstead, U.K.) replacing the 2% Type VII LGT agarose (Sigma Chemical Co., Poole, U.K.). Samples were run through a 1% pulsed-field certified agarose gel (Bio-Rad) at 180 V for 44 hours, with pulse times ramped from 6 to 72 seconds. For FAFLP, the selective primer combinations *Eco*+0 *Mse*+T, *Eco*+0 *Mse*+TA, and *Eco*+0 *Mse*+CG were used, and gel separation and fragment analyses were performed, all as described by Scott et al. (6).

All seven isolates had phage typing reactions corresponding to *S.* Typhimurium DT204b. This rare phage type was reported in only 40 cases in England and Wales in the 5-year period 1996–2000 and had never been identified in Iceland before this epidemic.

With the exception of resistance to nalidixic acid and decreased susceptibility to ciprofloxacin, the complete resistance spectrum (ACGKSSuTTm) was transferable to *E. coli* K12 rif^r^ as an intact linkage group. Plasmid profile analysis demonstrated the presence of five plasmids of 120, 65, 4.0, 3.0, and 2.0 MDa. Most transconjugants with the resistance pattern ACGKSSuTTm had a single incompatibility group H_2_ plasmid of 120 MDa. A few transconjugants had both the 120-MDa plasmid and an additional plasmid of 65 MDa. When PCR amplification was performed on DNA from transconjugants harboring either the single 120-MDa plasmid or both the 120-MDa and 65-MDa plasmids, positive results for *aad*A2, *bla*_TEM_, *sul1,* and *tet*A (class A) were obtained; *bla*_carb-2_, *tet*A (class G), and *tet*A (class B) were negative. When studied by integron PCR, one discrete 1.6-kb band was generated in transconjugants when either the 120-MDa plasmid or both the 120-MDa and the 65-MDa plasmids were present. In addition, one very faint amplicon of approximately 4 kb was consistently produced. Whether both the 120-Mda plasmid and the 65 Mda-plasmid have tetracycline resistance genes or whether tetracycline resistance is encoded by only the 120-MDa plasmid is unclear. When studied by GAMA, the *gyrA* mutation was that of aspartate to glycine (GAC-GGC) at codon 87.

PFGE profiles of all seven isolates were indistinguishable (Figure). To facilitate epidemiologic investigations, PFGE and plasmid profile TIFs of the banding patterns of these isolates and those of multiresistant *S.* Typhimurium DT204b from Scotland and Germany were exchanged electronically. In all cases, the resultant PFGE and plasmid profiles were indistinguishable. Finally, when studied by FAFLP, the seven isolates were identical.

**Figure:**
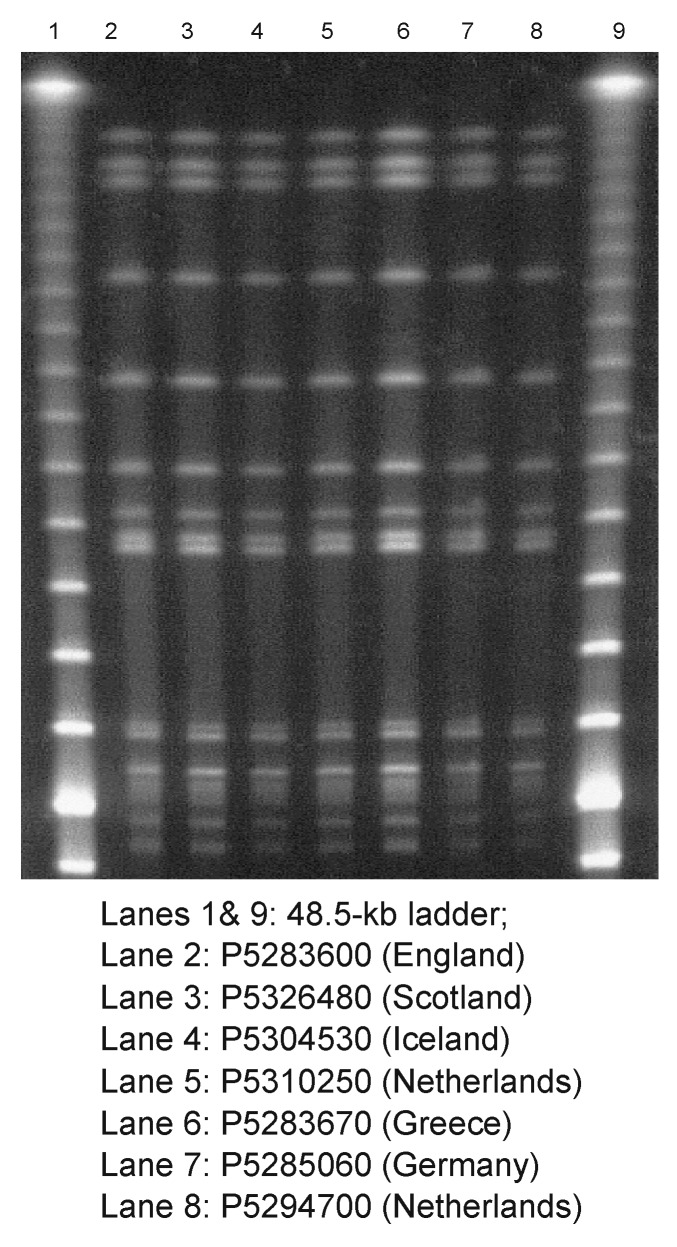
Pulsed-field gel electrophoresis profiles of *Xba*I-digested genomic DNA from isolates of *Salmonella* *enterica* serotype Typhimurium DT204b.

## Conclusions

These results confirm that the strains of *S.* Typhimurium DT204b of R-type ACGKSSuTTmNxCp_L_ responsible for outbreaks of infection in five European countries in the summer of 2000 were indistinguishable by all phenotypic and molecular criteria used for their characterization. A key aspect of this investigation was the rapid exchange of molecular fingerprints between laboratories already using standardized phage typing and antimicrobial susceptibility testing. In the United States, the exchange of molecular data has been addressed by the establishment of PulseNet, a national molecular typing scheme based on a standard method for PFGE; a similar network is being set up for the major Salmonella reference laboratories in Europe with research funding from the European Commission. Developing compatible networks for the exchange of real-time molecular data for *S. enterica* on an intercontinental scale would be of major benefit for the global control of salmonellosis.
